# Characterization of Mechanical and Hygroscopic Properties of Individual Canes of Reed

**DOI:** 10.3390/ma14092193

**Published:** 2021-04-24

**Authors:** Montaña Jiménez-Espada, Daniel Herrero-Adán, Rafael González-Escobar

**Affiliations:** 1 Department of Construction, School of Technology, University of Extremadura, Avda de la Universidad s/n, CP-10003 Cáceres, Spain; rgonzalegb@alumnos.unex.es; 2 Department of Engineering Design and Mathematics, University of the West of England, Bristol BS16 1QY, UK; Daniel2.Herreroadan@live.uwe.ac.uk

**Keywords:** reed, sustainable construction, ultimate stress, yielding stress, modulus of elasticity

## Abstract

The search for sustainability has led to the utilization of more ecological materials with at least, a similar structural performance to those used at present. In this regard, reed fits the environmental and structural requirements as it is a sustainable and biodegradable lignin-cellulose material with remarkable mechanical properties. This research confirms the reed’s structural efficiency as it demonstrates that it has excellent strength and stiffness in relation to its density. The reed anisotropy has a large impact on its properties. Indeed, the strength and stiffness parallel to the fibers are clearly higher than in the perpendicular direction. The results confirm that strength and stiffness decrease with the moisture content and nodes act as reinforcement in compression and bending. If compared with steel, timber and concrete, the reed possesses the highest value for strength. Hence, reed constitutes a strong candidate for environmentally friendly engineering.

## 1. Introduction

The common reed (*Phragmites australis* (Cav.) Trin. ex Steud., Poaceae) is one of the most widely distributed plant species on Earth, with a perennial growth form and aboveground organs that die back every winter [1]. It is a ubiquitous species in central Europe, colonizing a wide range of habitats, i.e., lake shores, fenland, brackish tidal marshes and dumping places, provided there is sufficient water in the subsoil. The height of the reed stalks varies from very short (0.5 m on inland saline soils) to very tall culms (5 m on fertile sediments of lakeshores and estuaries). However, even within a stand of limited area one can find remarkable variability in the performance of the individual stalks [2].

Similar to timber [3,4], reed consists of bundles of fibers that run parallel to the axis of the stem. Their cohesion is mainly governed by secondary bonds between these fibers, which lead to much stronger properties along the axial direction than in the transversal. In this type of grass, the cane is larger in diameter at the root than at the top and it is partitioned by nodes [5].

The use of reed as a renewable, organic construction material has recently caught the attention of researchers due to the topicality of energy saving and sustainable construction methods. In construction, reed has mainly been used as a material for covering roofs. Since the first half of the 20th century, reed has also been used as a lath board under plaster, and since the late 1930s, also for manufacturing reed insulating boards.

The surface of the reed stem does not absorb water or moisture due to its covering of silicon. Thus, the reed can be used for additional insulation in timber and log houses, where it provides one of the main benefits—vapor and air diffusion through the walls [6]. A well-known use of this stem, or cane, is in hut roofs and claddings for bioclimatic housing [7].

As an industrial material, reed can be used as a component in the manufacture of medium-density fiberboard [8] and as a source of polymers [9].

Reeds have also been used together with similar materials such as bamboo for shuttering, houses party walls or as a base for plastering (thin mats of reed) in internal partitions [10]. They also have excellent resistance to dynamic loads (earthquakes) due to their ductility and high resistance/self-weight ratio.

One of the most recent applications of this cane is for insulation acoustic panels [11]. Reed presents high sound absorption and performs well as an airborne noise insulator. Therefore, it is an ideal material for construction use [12]. Another application could be as reinforcement for concrete. Some studies were developed for bamboo [13,14,15,16] proving a reasonably good performance for certain applications.

A preliminary study on the bending elastic behavior of Hungarian reed stems showed that the mean value of the flexural stiffness obtained for reed stems was comparable to other studies related to reeds and performed in Japan [2]. Nevertheless, Hungarian reeds have a lower Young’s coefficient than bamboo, but it is similar to oak and beech (hardwoods) [17]. Plants with hollow, tube-like stems, such as *Phragmites australis*, combine a relatively high stiffness with a low biomass investment. However, hollow stems carry a risk of buckling. This risk might be reduced in reeds by the nodal stabilization in the stem [18].

Additional data for reeds can be found in the literature. Significant investigations related to the mechanical resistance of Phragmites stands were performed in [18,19,20]. Indeed, the elasticity of cane reeds was measured in previous studies using a three-point bending testing setup in order to obtain the mechanical properties along the direction of the reed fibers and in the orthogonal direction [21].

In relation to previous knowledge on the possible cyclic fatigue behavior of reed, [22] indicated that many plant materials show a higher stiffness on the second and subsequent cycles of load. Studying the cyclic-loading behavior of living plants in particular, demonstrates that it is not just polymer remodeling that causes this type of differential material behavior in cyclic loading, but the cellular responses also alter the cell wall deposition and composition. In [23], the flexural fatigue behavior of bamboo strips under different loading configurations was evaluated. The resulting gradient distribution of the vascular bundles along the thickness direction was mainly responsible for the exhibited anisotropic fatigue response, including fatigue life, hysteresis loops and residual stiffness. The investigation in [24] revealed that bamboo displays fatigue when loaded perpendicular to the fibers, but not when loaded parallel to the fibers.

Experiments with heat treatment have demonstrated that drying Phragmites samples at 120–160 °C increases their flexural strength and the technical elasticity limit [25]. This finding might be of interest when designing products derived from reeds, such as in [26], where the good parameters obtained for lightweight concrete blocks along with rising industrial demand for cost effective, lightweight and robust insulating materials suggest that Phragmites have great potential as feedstock for building materials.

This paper provides the mechanical properties of the individual reed canes and characterizes their hygroscopic behavior, i.e., variations in dimensions due to changes in the water content. Although, in reality reed canes are used in combination with other materials (plaster, concrete, bamboo or other lignocellulosic materials,) to obtain construction products, the properties of the individual canes are necessary to facilitate the design of those products. The paper is structured as follows. Section 2 describes the material and methods used to obtain the mechanical and hygroscopic properties. In Section 3 the results are presented along with a discussion. Section 4 concludes the work with the most relevant findings.

## 2. Materials and Methods

**Figure 1:**
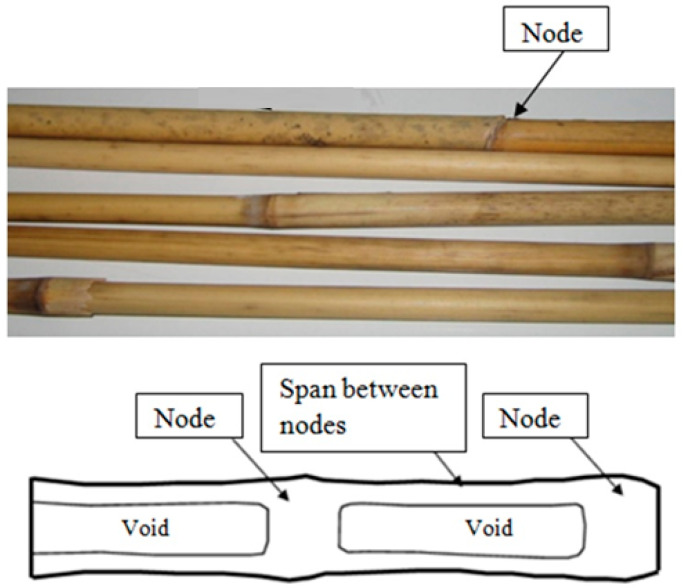
General view and longitudinal section scheme [19].

There are several types of *Phragmites australis*, which are different in terms of growth, location and properties. A Hungarian corporation, “Fertö-tavi Nádgazdasági Részvénytársaság” provided the materials employed in the laboratory experiments in different configurations (panels, bundles and rolls). Individual canes were extracted in order to develop the tests, which were inspired by the standards used in the case of structural timber, UNE-EN 408:2011 [27] and UNE-EN 384:2016 [28] As shown in Figure 1, a cane may be seen as a row of hollow cylindrical elements jointed by nodes. The dimensions used were the external diameter ($D_{e}$) and the wall thickness (t).

**Figure 2:**
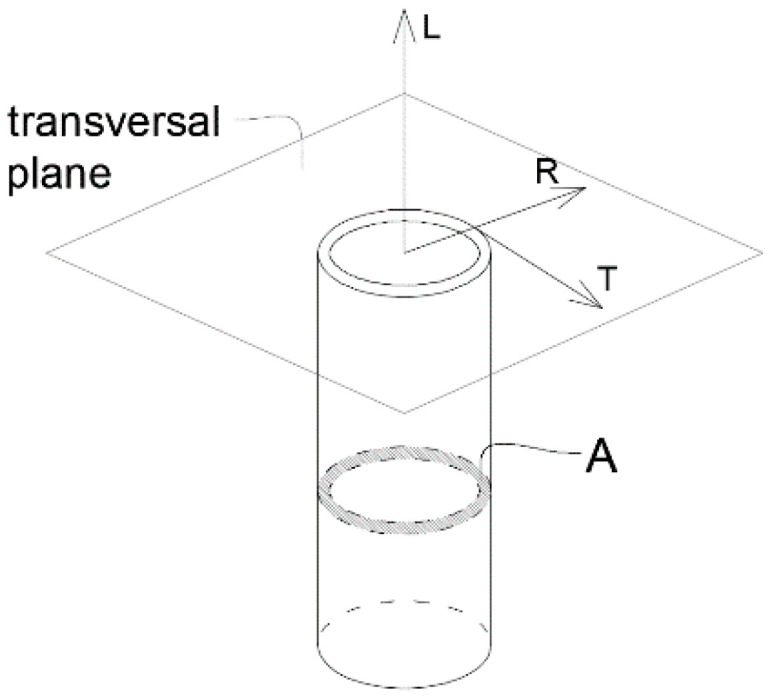
Directions of the cane and cross-sectional area.

Typically, the directions of study in canes are: longitudinal (L), radial (R) and tangential (T), as illustrated in Figure 2, where the cross-sectional area is also represented. In this work, the focus was on the longitudinal direction since it is the strongest orientation and the one used for construction purposes.

Three types of mechanical tests were carried out: compression, bending and tension. The three tests were applied to specimens with and without nodes in order to study the influence of those. Ultimate stress, yielding stress and modulus of elasticity were calculated for each type of load. The inputs for these calculations came from the universal testing machine at the Forest Products Industries Teaching Unit of the Technical School of Forestry, which provided the data points for the load–displacement curve. These data were used for subsequent calculations.

Different parts connected to the machine bridge allowed for different tests: dices for compression, a bending bench and loading cylinder for bending and clamps for tension. The loading rate and the time to mechanical failure is shown in Table 1.

Apart from the mechanical tests, the dimensional change versus humidity content was also measured. In order to determine the anhydrous weights and calculate the moisture content, the specimens were dried at 103 ± 2 °C using a SELECTA programmable electric oven with temperature regulation between 40 and 250 °C according to UNE-EN 13183-1:2002 [29].

The humidity content of one specimen (*h*) was calculated as per Equation (1), which finds the percentage of water mass in the specimenin comparison to the fully dried, or anhydrous mass ($m_{0})$.

(1) $$h\left(m\;\%\right)=100\frac{m_{h}-m_{0}}{m_{0}}$$

The specimen was weighed on a scale ($m_{h}$), then it was placed in a drying chamber at 103 °C to eliminate the water. The specimen was extracted from the chamber and weighed until the value was constant, indicating that the anhydrous mass was reached ($m_{0})$. This methodology is well known in the timber industry [3,4].

Three humidity content values were considered:

1. Sample in the heating unit: the nominal humidity content in the material was 7 m%;
2. Room conditions: the nominal humidity content in the material was 15 m%;
3. External humidity conditions: the nominal humidity content in the material was 90 m%.

These three ambient conditions were used for testing compression and bending. However, for tension only the second condition were applied due to the complexity of the test procedure. The average humidity in this case was 15 m%.

The selected canes were undamaged (no fissures, dents or rottenness). The number of specimens tested is given in Table 2.

### 2.1. Compression Test

The length of the samples was between 19 and 35 mm and the external diameters between 4 and 7 mm such that the slenderness was 25 or less in order to avoid compression buckling. The average length, external diameter and thickness of all the compression samples was 27 mm, 5.7 mm and 0.79 mm, respectively.

**Figure 3:**
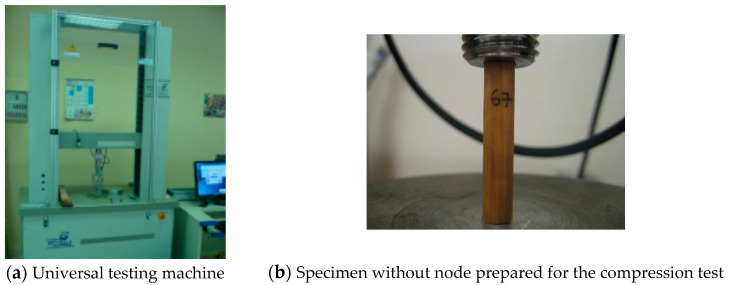
Image of the universal testing machine (**a**) and example of specimen in the compression tests (**b**).

The specimens were compressed until crushing failure. Figure 3 shows the setup of the test and the universal testing machine employed.

**Figure 4:**
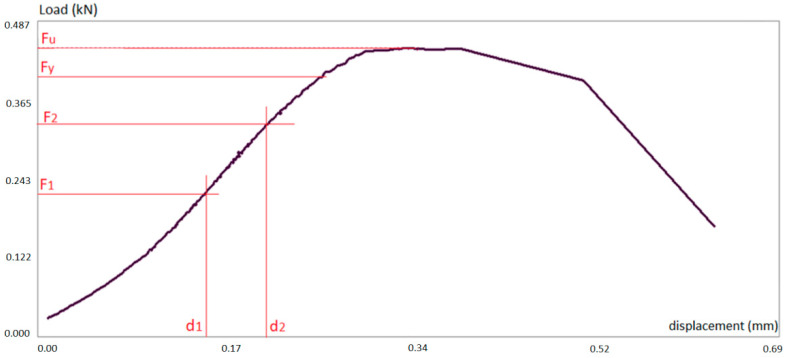
Load–displacement plot for compression test.

Typically, the load–displacement curve followed the pattern illustrated in Figure 4. The displacement refers to the top metal dice of the testing machine. The curve shows the first settlement phase where the clearance of the testing machine joints are adjusted and the tips of the specimen are locally crushed, achieving fully contact against the machine dices. Secondly, the load increases linearly with the displacement in the elastic stretch. At this part, two points were selected for the calculation of the modulus of elasticity. Once the elastic limit load is achieved ($F_{y}$), the material behaves plastically until the maximum load is reached ($F_{u}$). Afterwards the specimen loses its mechanical integrity and the load decreases strongly, leading to the collapse of the sample.

For each specimen, the ultimate stress, yielding stress and elastic modulus were calculated as per Equations (2)–(4), respectively. For further details on these equations refer to [18].

(2) $$S_{uc}=\frac{F_{u}}{A}$$

(3) $$S_{yc}=\frac{F_{y}}{A}$$

(4) $$E_{c}=\frac{\frac{F_{2}-F_{1}}{A}}{\frac{d_{2}-d_{1}}{L}}$$

where:

$F_{u}$ is the maximum or ultimate supported load;

$F_{y}$ is the load at the yielding limit;

$F_{1}$ and $F_{2}$ are the lower and upper loads measured in the elastic range;

$d_{1}$ and $d_{2}$ are the lower and upper displacements measured in the elastic range;

A is the cross-sectional area of the specimen;

L is the initial length of the specimen.

If we recall Figure 4 where these forces and displacements are represented, the yielding limit force ($F_{y}$) is that one that produces the remaining displacement equal to 1% of the initial length of the specimen.

**Figure 5:**
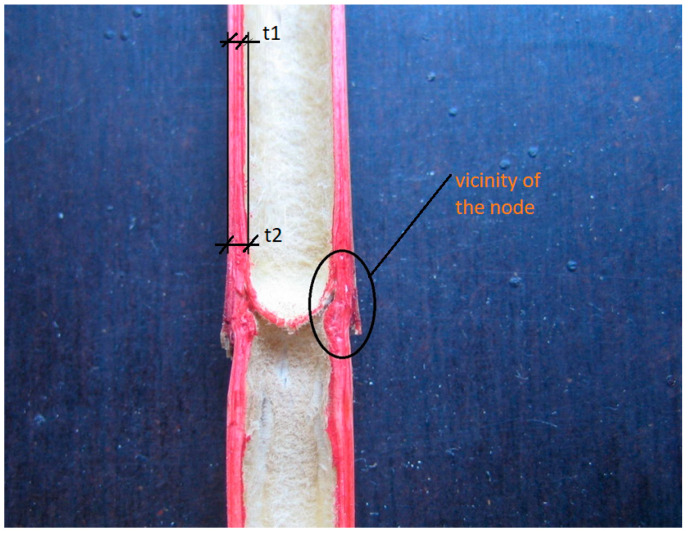
Longitudinal section of reed stem with node, where the wall is thicker in the vicinity of the node (t2) against the rest of the stem (t1).

For specimens with nodes, the calculated elastic modulus is overestimated because the calculations do not consider the thickening of the walls around the node. As illustrated in Figure 5, the wall at the node location is thicker. This results in extra stiffness that is not considered in the calculation. A rigorous methodology should assume a variable cross-sectional area and solve the elastic modulus by integration.

### 2.2. Bending Test

**Figure 6:**
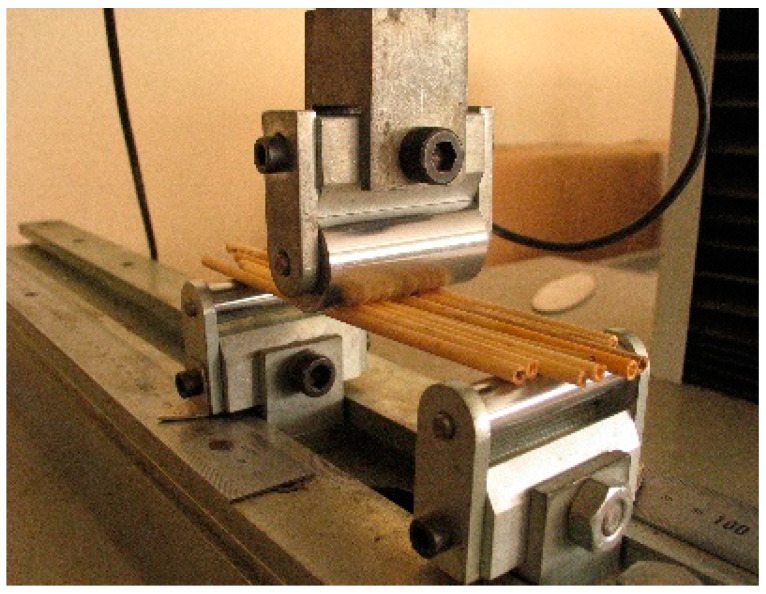
Setup for bending testing.

The three-point bending test was carried out on stems with and without nodes, as illustrated in Figure 6. The span was 130 mm, hence the sample lengths had to be longer, typically 140 mm or more. Cane diameters were between 3.40 and 8.75 mm. The average external diameter and thickness of all the bending samples was 5.8 mm and 0.76 mm, respectively.

The canes were grouped according to their diameter with a maximum variation of 8% over the group diameter average. The number of canes per group varied from 3 (larger diameters) to 8 (smaller diameters). Each group was fixed on the bending bench to form a plane such that all the specimens together supported the load from the loading cylinder (see Figure 6). The total inertia is the addition of the inertia of each cane. A certain amount of reed crushing at contact with the loading cylinder was unavoidable, therefore the bending stiffness results are slightly lower than the real value. The specimens were bent until failure.

The typical load–deflection curve followed a pattern similar to the compression test (see Figure 4), with three stretches: settlement, elastic and plastic until failure.

For each specimen, the ultimate stress, yielding stress and elastic modulus were calculated as per Equations (5)–(7), respectively. Ultimate stress is computed under linear elastic assumptions while in reality the material yields. However, for comparison purposes this is considered acceptable. For further details on these equations refer to [30].

(5) $$S_{uf}=\frac{F_{u}\;L\;}{4\;W}$$

(6) $$S_{yf}=\frac{F_{y}\;L\;}{4\;W}$$

(7) $$E_{f}=\frac{\left(F_{2}-F_{1}\right)\;L^{3}\;}{48\;\left(f_{2}-f_{1}\right)\;I}$$

where:

$F_{u}$ is the maximum or ultimate supported load;

$F_{y}$ is the load at the yielding limit;

$F_{1}$ and $F_{2}$ are the lower and upper loads measured in the elastic range;

$f_{1}$ and $f_{2}$ are the lower and upper displacements (deflections) measured in the elastic range;

L is the span, in this case 130 mm;

I is the inertia of the set of canes, computed as (8).

W is the elastic modulus of the set of canes computed as (9);

(8) $$I=\overset{n}{\underset{i=1}{\sum }}\left[{\left(\frac{De_{i}}{2}\right)}^{4}-{\left(\frac{Di_{i}}{2}\right)}^{4}\right]\cdot \frac{\pi }{4}\;$$

(9) $$W=\frac{I\;}{\frac{\sum_{i=1}^{n}r_{i}}{n}}$$

with n being the number of canes in the set, and $De_{i}$, $Di_{i}$ and $r_{i}$ the external diameter, internal diameter and external radius of the *i*th cane of the set. The yielding limit force ($F_{y}$) was the one that produces a remaining displacement equal to 5% of the total displacement of the test.

As per the compression test, the calculation of the elastic modulus of the canes with nodes was overestimated since the increment in the walls’ thickness at the nodal zones was ignored.

### 2.3. Tension Test

The samples’ length and diameter varied between 120 and 220 mm and 4 and 7 mm, respectively. The average length, external diameter and thickness of all the tension samples was 120 mm, 5.7 mm and 0.86 mm, respectively.

**Figure 7:**
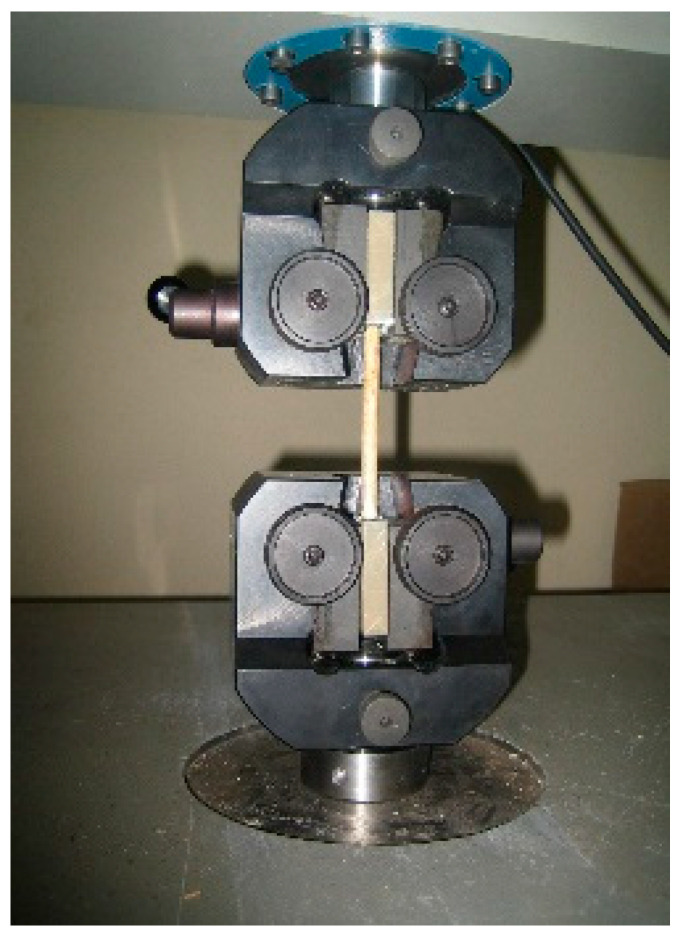
Setup for tensile test.

The tips’ surfaces were sanded in order to remove the natural silicon coat of reed and increase the friction coefficient. Both of the ends were embedded in an epoxy resin cast. Once the resin was cured, the sample could be fixed to the tension clamps (see Figure 7), avoiding any crushing to the cane. The clear length between the epoxy resin supports was between 80 and 175 mm.

**Figure 8:**
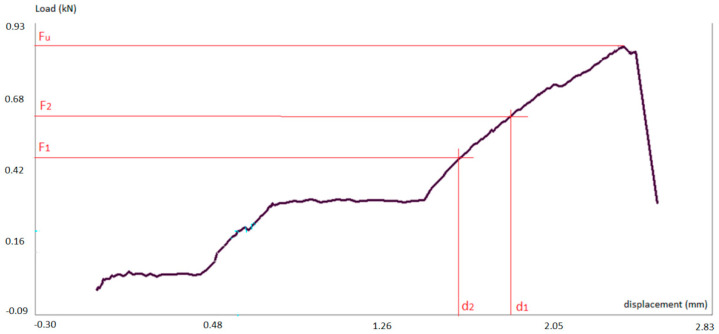
Load–displacement plot for tensile test.

Typically, the load–displacement curve followed the behavior plotted in Figure 8. Initially there is settlement stretch where one the clamps of the machine slides on the resin block until it is fully retained. Then the elastic stretch is initiated. A second settlement at the other clamp may occur during the elastic stretch. Apart from these two settlements, the behavior is linear until failure with no plastic stretch.

For each specimen, the ultimate stress and elastic modulus were calculated as per Equations (10) and (11), respectively. For further details on these equations refer to [18].

(10) $$S_{uc}=\frac{F_{u}}{A}$$

(11) $$E_{t}=\frac{\frac{F_{2}-F_{1}}{A}}{\frac{d_{2}-d_{1}}{L}}$$

where:

$F_{u}$ is the maximum or ultimate supported load;

$F_{1}$ and $F_{2}$ are the lower and upper loads measured in the elastic range;

$d_{1}$ and $d_{2}$ are the lower and upper displacements measured in the elastic range;

A is the cross-sectional area of the specimen;

L is the initial clear length (clear distance between resin blocks).

These forces and displacements are represented in Figure 8. The yielding and ultimate limits coincide in the tensile test.

### 2.4. Dimensional Change

The anhydrous density was obtained from the compression and bending specimens without nodes. The average and variation coefficient are:

(12) $$\rho_{o}=0.508\;g/{\mathrm{cm}}^{3}\;\;\;\;VC=16\%$$

**Figure 9:**
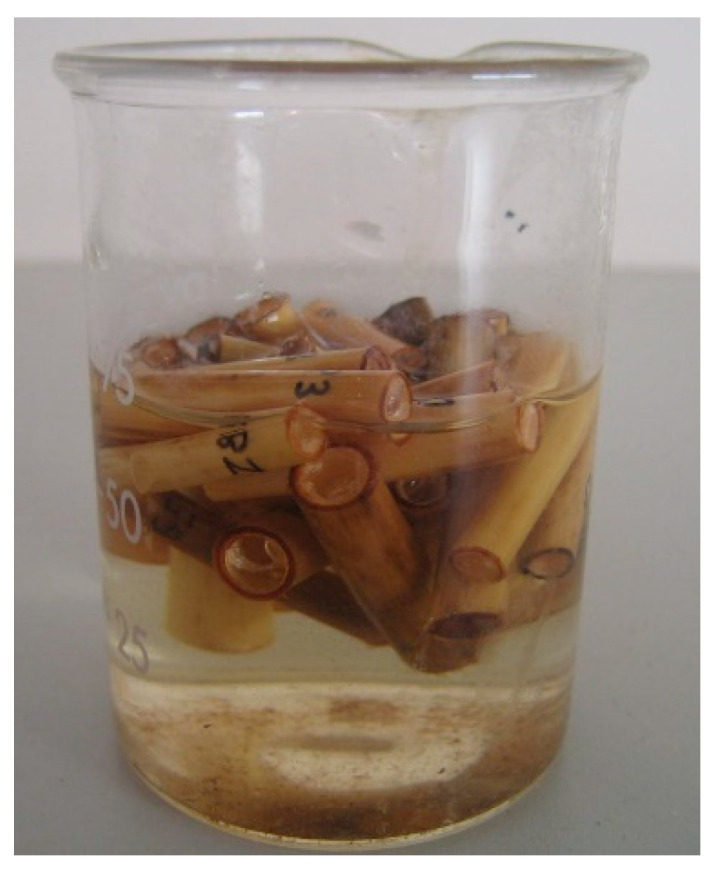
Immersion of specimens.

To characterize the dimensional changes versus humidity content, the external diameter ($D_{e}$) and thickness (t) were measured, with the moisture varying from fully saturated to zero humidity. The specimens were immersed in water for 24 h (see Figure 9). Then, they were extracted and the dimensions and mass were measured at two-hour intervals while the specimen was drying in room conditions in between. After 12 h, they were placed in the drying chamber to eliminate all the water (anhydrous conditions) and the dimensions were measured again. The anhydrous weight allowed us to compute the humidity in the previous states by the difference between them (see Equation (1)).

## 3. Results and Discussion

### 3.1. Dimensional Change due to Humidity

Table 3 provides the average dimensional changes: external diameter ($D_{e}$) and thickness (t) of the specimens from saturated to anhydrous humidity content. The dimensional changes are referred to as a percentage of the dimensions in anhydrous conditions. The total increment in moisture content was 83 m%.

**Figure 10:**
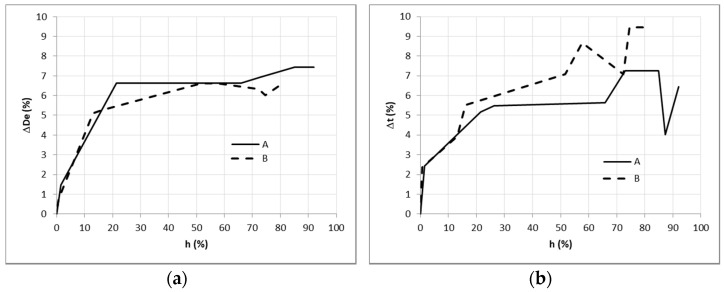
Evolution of external diameter (**a**) and thickness (**b**) against moisture content.

In addition, in two of the specimens, the evolution of the mentioned dimensions was recorded as illustrated in Figure 10 (the specimens are referred to as A and B). The increments are given as percentage of the anhydrous values. This figure shows similar behavior to timber, where two stretches are distinguished: from 0 to 20–30 m%, where most of the dimensional change happens, and above where the changes are much reduced.

Table 3 shows the variations equal to or less than 8%. The impact of these changes on the calculation of the mechanical properties was ignored due to its reduced value. The measurement of these dimensional changes was performed to characterize the hygroscopic behavior of the reed.

### 3.2. Strength and Stiffness

Table 4 gives the average values for yielding stress ($S_{y}$), ultimate stress ($S_{u}$) and elastic modulus (E) for each type of test. Stresses are rounded up to units and elasticity modules to hundreds. The lower figure is the variation coefficient as a percentage, VC=100 σ/x; where σ is the standard deviation and x the corresponding average.

**Figure 11:**
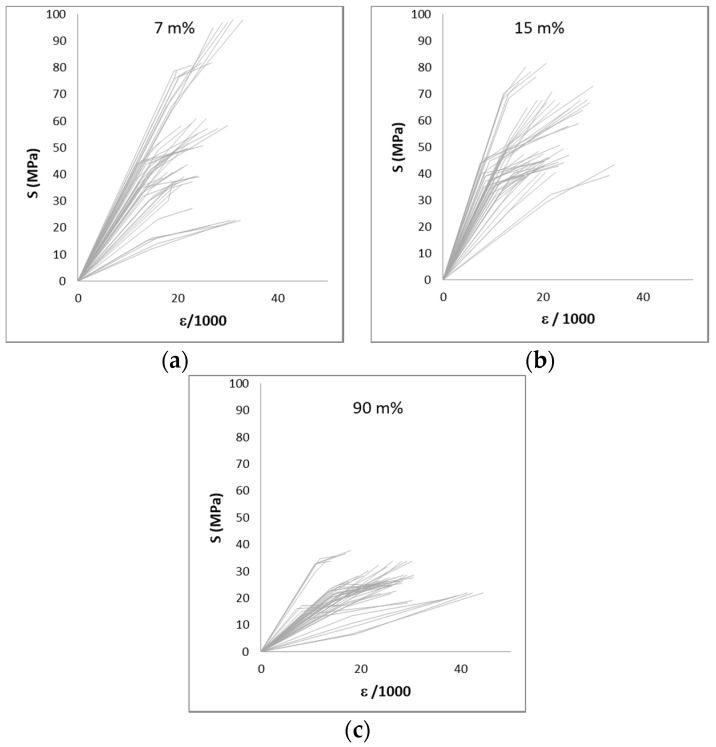
Stress–strain diagram for the compression of 50 samples without nodes. Ranges of humidity: 7 m% (**a**), 15 m% (**b**) and 90 m% (**c**).

**Figure 12:**
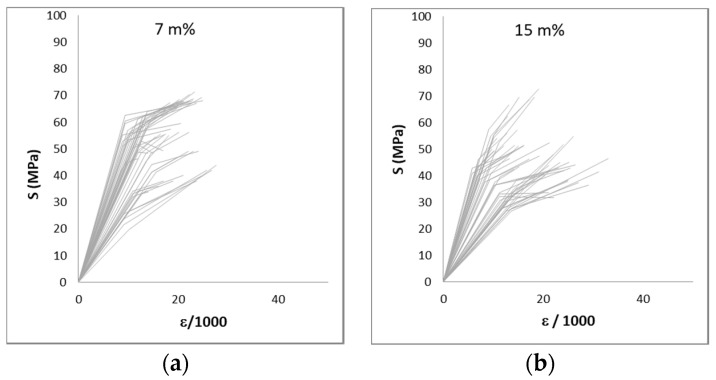

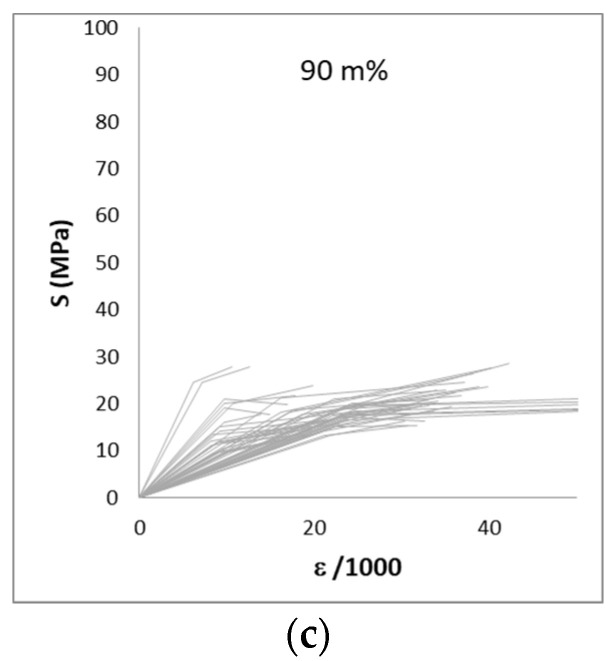
Stress–strain diagram for the compression of 50 samples with nodes. Ranges of humidity: 7 m% (**a**), 15 m% (**b**) and 90 m% (**c**).

**Figure 13:**
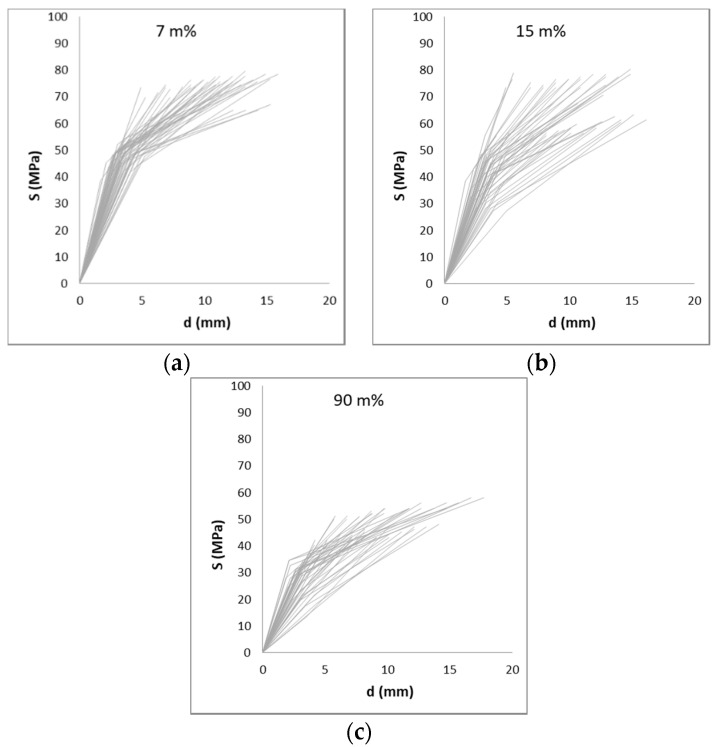
Stress–deflection diagram for the bending of 50 samples without nodes. Ranges of humidity: 7 m% (**a**), 15 m% (**b**) and 90 m% (**c**).

A wide range of variation in the results could be expected considering that reed is a natural product.. The coefficient of variation reaches values up to 69%, but more frequently, values approach 20–40%. Figure 11, Figure 12 and Figure 13 show the large variation in the results. Figure 11 and Figure 12 show the stress–strain diagrams for samples in compression. A stress–strain diagram was constructed for each sample, with its elastic and ultimate stresses ($S_{y}$ and $S_{u}$) and the recorded strain for both stresses measured with respect to the initial length of the sample. Figure 13 plots the results for the bending tests in samples without nodes. In this latter case, a stress–deflection diagram is presented.

### 3.3. Nodes’ Influence

**Figure 14:**
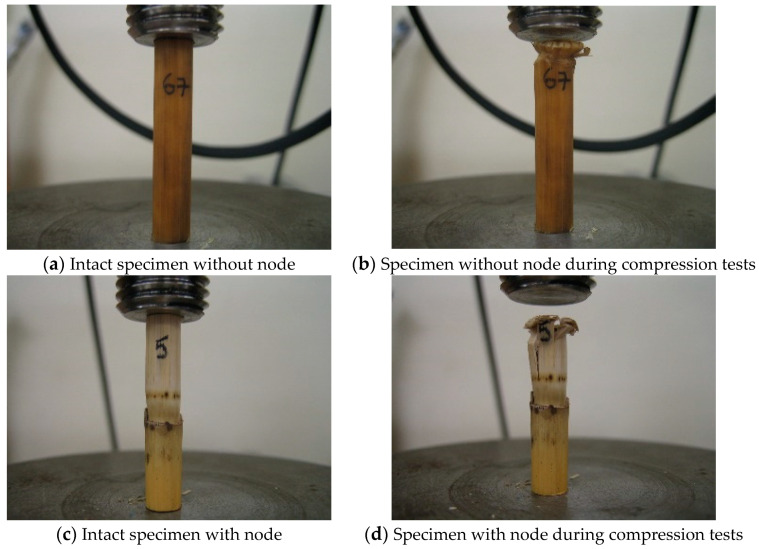
Compression tests in specimens without node (**a**,**b**) and with node (**c**,**d**).

In the compression tests, the yielding and ultimate stress limits are very similar for the specimens with and without nodes. Figure 14d shows that the failure in specimens with nodes happens in the part without a node. It can be observed in Figure 8 that the failures for specimens with and without nodes are similar. Specimens with nodes tested to bending failed at locations that were outside the nodes influence. Therefore, it seems that nodes have no influence on the strength of the canes since they fail at the locations with no nodes. The nodes do indeed give the cane a strong core (failure does not happen at the node), which contributes to the larger stiffness, but they are not able to strengthen the whole cane.

Regarding the tension tests, the node strengthening was not so clear. On one hand, the ultimate stress was larger in specimens with nodes (94 MPa versus 64 MPa as shown in Table 4). On the other hand, 40% of the specimens tested failed at the node location, which may indicate a weak point at the nodes. In order to clarify this question, the development of additional tests would be necessary.

Nodes’ effects were studied on a similar material, bamboo, which was subjected to tension and compression tests [31]. The presence of nodes increased the compression strength and stiffness and decreased the tension performance of bamboo.

### 3.4. Moisture Content Influence

**Figure 15:**
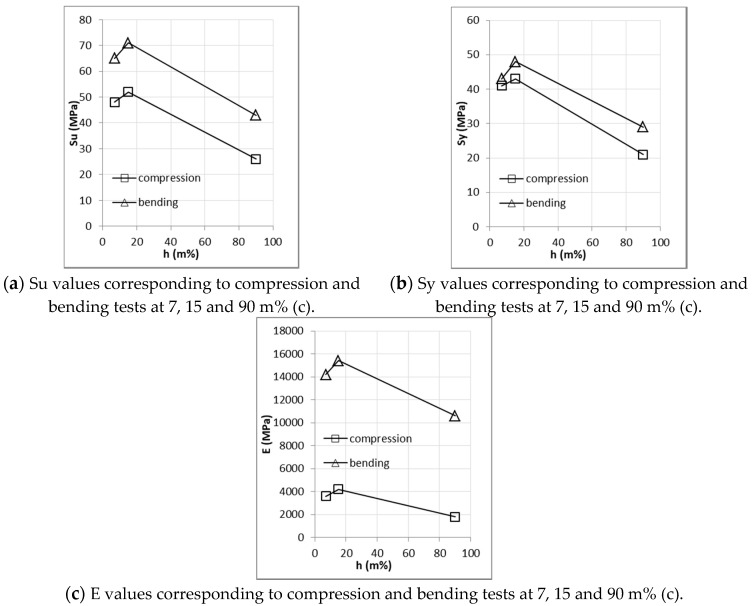
Variation in mechanical properties against humidity content for compression and bending samples without nodes (**a**–**c**).

Figure 15 presents the results from the compression and bending tests without nodes. This figure shows clearly that higher moisture content reduces the mechanical properties: both strength and stiffness are practically halved in the specimens corresponding to 90 m% compared to the drier specimens (7 m% and 15 m% of moisture content).

In contrast, the behavior of the mechanical properties of specimens with 7 m% and 15 m% of moisture did not show a clear tendency, despite showing a slight improvement in the 15 m% specimens.

### 3.5. Density Influence

**Figure 16:**
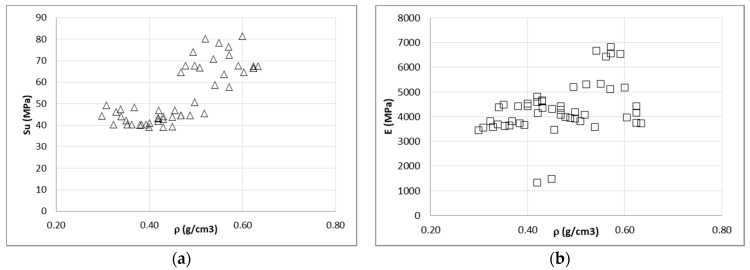
Ultimate stress (**a**) and elastic modulus (**b**) against density.

There is slight increasing tendency for the mechanical properties as the density increases but the scattered data are not definitive. As an example, Figure 16 shows the mechanical properties in compression against density for the 15 m% moisture content of the 50 specimens.

### 3.6. Comparison with Steel, Concrete and Timber

Using compression as a reference value, reeds (15 m% moisture with no nodes) were compared with construction materials used nowadays. Steel, timber and concrete were selected from the Eurocodes [32,33,34].

Since the Eurocodes use characteristic values instead of average values for the yielding stress limits, we needed to calculate the characteristic yield limit of the reed (Sy_k_) by deducting the variance to the average as follows:

Sy_ave_ = 43 MPa, σ = 10.75 MPa => Sy_k_ = 32 MPa (13)

Table 5 summarizes the comparison of the calculated specific strengths and stiffness, i.e., the values divided per kg of mass.

It can be noticed in Table 5 that reed possesses the highest value for specific strength.

Taking into account the previous investigation and arguments, it can be concluded that reed constitutes a strong candidate for environmentally friendly engineering and presents remarkable specific mechanical properties.

In the author’s opinion, it would be very appropriate to develop more advanced research including tests such as cyclic fatigue testing of the material. In addition, future lines of research by the correspondence author include durability, acoustic and thermal tests in order to characterize all the properties of reeds for construction purposes, similar to [35,36]. Previous research carried out by the author confirmed the suitability of reeds for use in acoustic screens for roads and offered proposals for new acoustically-efficient construction elements for buildings, such as in the investigation conducted in [37].

## 4. Conclusions

The aim of the research carried out in this article was the estimation of the mechanical properties of individual reed canes from Fertö lake and the characterization of their hygroscopic behavior. It can be concluded that the mechanical properties of reeds are acceptable for certain lightweight construction since the material possesses a high specific strength and an acceptable specific stiffness compared to other typical materials.

The results highlight that the properties decline as the moisture content increases, a feature shared with timber. Both materials are hygroscopic lignin-cellulosic, and hence, are influenced by the water located inside their microstructure.

Nodes do not strengthen the cane as a whole element, although they provide a stiff and high strength core. In tension, nodes might be a point of weakness. Regarding the density, the material shows a slight tendency to be stronger and stiffer as the density increases.

The application of reeds in construction does not involve the use of the canes themselves, but they are combined in bundles or panels, or with other materials, e.g., timber products, plaster or concrete. However, the characterization of the individual canes carried out in this paper facilitates the future development of the aforementioned construction products.

## Figures and Tables

**Table 1:** Loading rate and time until failure for each type of test.

	Type of Load		
	Compression	Bending	Tension
Loading Rate (N/s)	10	3	15
Test Time (s)	60 ± 30	60 ± 30	60 ± 30

**Table 2:** Number of specimens tested.

	*h* (m%)	Type of Load		
		Compression	Bending	Tension
Specimen without nodes	7	50	50	-
	15	50	50	50
	90	50	50	-
Specimen with nodes	7	50	50	-
	15	50	50	50
	90	50	50	-

**Table 3:** External diameter (De) and thickness (t) variations.

Δ*h* (m%)	Δ*D_e_* (%)	Δ*t* (%)
83	6.1	8.0

**Table 4:** Summary of mechanical properties.

		Compression			Bending			Tension	
	h (m%)	Sy (MPa)	Su (MPa)	E (MPa)	Sy (MPa)	Su (MPa)	E (MPa)	Su (MPa)	E (MPa)
No node	7	41 _43_	48 _45_	3600 _40_	43 _6_	65 _9_	14,200 _17_	-	-
	15	43 _25_	52 _23_	4200 _30_	48 _15_	71 _11_	15,400 _19_	64 _34_	2200 _49_
	90	21 _21_	26 _17_	1800 _33_	29 _8_	43 _8_	10,600 _24_	-	-
With node	7	46 _25_	51 _25_	4600 _22_	-	91 _16_	13,300 _15_	-	-
	15	39 _26_	46 _23_	4700 _55_	-	70 _6_	10,900 _4_	96 _21_	6000 _23_
	90	16 _26_	19 _25_	1700 _69_	-	49 _8_	12,200 _8_	-	-

**Table 5:** Comparison of specific compression strength and stiffness.

Material	Sy_k_ (MPa)	E (MPa)	Density ρ (kg/m^3^)	Specific Strength (N·m/kg)	Specific Stiffness (kN·m/kg)
Reed	32	4200	508	63.0	8.3
Steel S275	275	210,000	7850	35.0	26.8
Timber C16	17	8000	370	45.9	21.6
Concrete C25	33	31,000	2350	14.0	13.2
